# Vison transformer adapter-based hyperbolic embeddings for multi-lesion segmentation in diabetic retinopathy

**DOI:** 10.1038/s41598-023-38320-5

**Published:** 2023-07-10

**Authors:** Zijian Wang, Haimei Lu, Haixin Yan, Hongxing Kan, Li Jin

**Affiliations:** 1 School of Medicine and Information Engineering, Anhui University of Chinese Medicine, Hefei, 230012 China; 2 School of Basic Medical Sciences, Anhui Medical University, Hefei, 230032 China; 3 Hefei University of Technology, Hefei, 230009 China

**Keywords:** Mathematics and computing, Diseases

## Abstract

Diabetic Retinopathy (DR) is a major cause of blindness worldwide. Early detection and treatment are crucial to prevent vision loss, making accurate and timely diagnosis critical. Deep learning technology has shown promise in the automated diagnosis of DR, and in particular, multi-lesion segmentation tasks. In this paper, we propose a novel Transformer-based model for DR segmentation that incorporates hyperbolic embeddings and a spatial prior module. The proposed model is primarily built on a traditional Vision Transformer encoder and further enhanced by incorporating a spatial prior module for image convolution and feature continuity, followed by feature interaction processing using the spatial feature injector and extractor. Hyperbolic embeddings are used to classify feature matrices from the model at the pixel level. We evaluated the proposed model’s performance on the publicly available datasets and compared it with other widely used DR segmentation models. The results show that our model outperforms these widely used DR segmentation models. The incorporation of hyperbolic embeddings and a spatial prior module into the Vision Transformer-based model significantly improves the accuracy of DR segmentation. The hyperbolic embeddings enable us to better capture the underlying geometric structure of the feature matrices, which is important for accurate segmentation. The spatial prior module improves the continuity of the features and helps to better distinguish between lesions and normal tissues. Overall, our proposed model has potential for clinical use in automated DR diagnosis, improving accuracy and speed of diagnosis. Our study shows that the integration of hyperbolic embeddings and a spatial prior module with a Vision Transformer-based model improves the performance of DR segmentation models. Future research can explore the application of our model to other medical imaging tasks, as well as further optimization and validation in real-world clinical settings.

## Introduction

Diabetic retinopathy is an ocular condition resulting from injury to the retina caused by hyperglycemia in individuals with diabetes^1^. The retina is a delicate membrane located at the posterior of the eye that converts light into electrical impulses transmitted to the brain^2^. Diabetic retinopathy is classified into two categories: Non-Proliferative DR (NPDR) and Proliferative DR (PDR)^3^. NPDR is characterized by damage to the small blood vessels in the retina, potentially resulting in blood or fluid leakage^4^. PDR is defined by the growth of abnormal blood vessels on the surface of the retina, which can lead to intraocular bleeding, potentially causing retinal detachment and vision loss^5^. It is important for individuals with diabetes to undergo regular eye exams for early detection and appropriate treatment of DR^6^. The increasing prevalence of DR globally highlights the urgent need for automated diagnostic tools to enhance the early detection and prevention of DR^7^. Various deep neural networks have been employed for studying the diagnosis of DR, specifically in terms of feature extraction, image classification, and lesion detection and segmentation^8–12^. Deep neural networks have been shown to be effective in medical images analysis, particularly in detecting abnormalities and lesions^13–16^. By leveraging these techniques, researchers aim to improve the accuracy and efficiency of DR diagnosis^17^.

In this paper, the utilization of the most recent and widely acknowledged deep learning technique was employed to enhance the precision of DR lesion segmentation, specifically the Transformer-based model. Recently, the efficacy of the Transformer-based model has demonstrated a trend of surpassing that of convolutional neural networks and provides state-of-the-art performance in natural image classification, detection, and segmentation^18^. However, it still needs further research in the application of deep learning techniques to medical image processing^19^. Researchers have pointed out that hyperbolic space may exhibit superior performance compared to the traditional neural network based on Euclidean space^20^. Therefore, hyperbolic embeddings were introduced in this paper to conduct the pixel-level classification, and the experimental results demonstrate that hyperbolic space can enhance the performance of the model. The following are the contributions of this work:

- We propose a Transformer-based model named “VTA (Vision Transformer Adapter) + HBE (Hyperbolic Embeddings)” suitable for DR multi-lesion segmentation tasks. Our approach adapts the original Vision Transformer model by incorporating a spatial prior module, which leverages convolutional neural networks to extract image features. The architecture also incorporates the spatial feature injector and extractor to enhance feature interaction.
- We employ hyperbolic embeddings to classify the feature representation at the pixel level. This solution effectively addresses the challenges encountered in current implementations of hyperbolic polynomial logistic regression, resulting in a more efficient parallel pixel classification method for image segmentation tasks.
- The proposed model demonstrates superior performance in the segmentation of hard exudates and microaneurysms on the IDRiD dataset, and exceptional performance in the segmentation of microaneurysms and soft exudates on the DDR dataset.

## Related work

### Deep neural networks in DR lesion segmentation

Despite Deep Neural Networks (DNNs)^21,22^ being prevalent in the segmentation of DR lesions, they still face two major challenges: significant morphological variations of DR lesions across different degrees and difficulty distinguishing DR lesions from similar structures^23^. The challenge in utilizing deep learning models for detecting small-size DR lesions lies in their ability to differentiate between normal and abnormal features. The limited size of these lesions, sometimes consisting of only a few pixels, can significantly impact the model’s accuracy and hinder its ability to make reliable predictions.

Researchers have put forth numerous proposed enhancements and improvements to address the aforementioned challenges. Zhang et al.^24^ present a feature fusion algorithm that leverages a multilayer attention mechanism for improved feature layer and channel fusion. The proposed method allows for more accurate selection of feature layers containing small target features, leading to improved preliminary detection of small targets. The findings of the study highlight the efficiency of the algorithm, resulting in a noteworthy increase in the average accuracy and sensitivity of microaneurysms detection. Wang et al.^25^ introduced a semi-supervised collaborative learning model to enhance the precision of DR grading and lesion segmentation. The model leverages attention mechanism technology and utilizes low-level guidance to identify lesion features and high-level guidance to create lesion attention images. These attention images serve as pseudo masks to facilitate the training process of the segmentation model. Xie et al.^22^ propose a novel and versatile framework to improve the precision of existing deep convolutional neural networks for medical image segmentation (including DR multi-lesion segmentation).

### Hyperbolic deep learning

DNNs are characterized by their multi-layer hybrid structure and multiple residual connections, allowing for the potential to model complex functions theoretically and leading to their dominance in research areas such as image classification and segmentation. Neural architectures based on Euclidean space are optimized primarily for raster data, which limits their ability to effectively handle optimization problems involving structured data in non-Euclidean spaces. Its relying on local proximity can result in the incorrect representation of geometric structures and undermine the effectiveness of these architectures for such tasks. The Hyperbolic deep learning has gained widespread attention for its ability to effectively represent tree-like structures, taxonomies^26,27^, text^28,29^, and graph data^30,31^. Researchers have put forth a number of hyperbolic alternatives for network layers that span from intermediate to classification layers^20,32,33^.

A recent study by^34^ has expanded the utilization of hyperbolic space for semantic image segmentation. The research team has reformulated the hyperbolic multinomial logistic regression approach to ensure tractability. The finding of using hyperbolic space in semantic image segmentation can benefit with higher proficiency, such as enhanced zero-shot generalization and improved performance in low-dimensional embeddings. Ganea et al.^32^ bridge the gap between hyperbolic and Euclidean geometry in neural networks and deep learning, opening new possibilities in Geometric Deep Learning (GDL). They achieve this by generalizing basic operations, multinomial logistic regression, feed-forward, and gated recurrent neural networks to the Poincaré model of hyperbolic geometry using gyrovector spaces and generalized Möbius transformations. They introduce a unified framework that smoothly parametrizes basic operations and objects in constant negative curvature spaces and demonstrate how Euclidean and hyperbolic spaces can be transformed into each other. The effectiveness of hyperbolic neural network layers is demonstrated through experiments on textual entailment and noisy-prefix recognition tasks.

### Transformer in medical images

Vaswani et al.^35^ introduced Transformers architecture, a transformative design that features encoders and decoders as its fundamental components. The encoders employ attention mechanisms to consolidate information from input sequences into high-dimensional representations, while decoders are employed to extract these high-dimensional representations to generate target sequences. Since their inception, Transformer-based architectures have established a strong track record of delivering state-of-the-art performance on a variety of natural language processing and computer vision tasks. The success of Transformers is attributed to its highly parallelizable, which allows for efficient training on large datasets and fast inference during deployment and captures context effectively^36^. The impact of Transformers on the field of AI continues to be significant, with ongoing research aimed at further improving their performance and exploring their applications in new domains.

Recently, the inception of the Vision Transformer model has sparked a trend in the medical image processing community towards the adoption of Transformer-based architectures or hybrid convolutional neural networks to enhance model performance^19^. Shen et al.^37^ proposed a novel convolution-and-transformer network, which is built on the encoder-decoder architecture and demonstrates efficacy in the segmentation of kidney cysts. Wang et al.^38^ were pioneers in applying Transformer-based architecture for the efficient 3D segmentation of brain tumors. Their network leverages the encoder to extract volumetric spatial features, which are then transmitted to the decoder for upsampling and the generation of a full-resolution segmentation map. Yun et al.^39^ proposed a novel Spectral Transformer model for the segmentation of hyperspectral pathology images. The model leverages a sequence-to-sequence prediction procedure to facilitate the learning of contextual features from spectral bands, thus enabling more accurate and efficient segmentation. Despite their remarkable performance in various applications, there remains potential for further exploration and development of Transformers in the field of medical imaging.

## Hyperbolic space

### Poincaré ball model

The Poincaré ball model, named after the mathematician Henri Poincaré, is a mathematical representation of hyperbolic geometry. We utilize Euclidean concepts such as distance and angle to reason about hyperbolic spaces, making it an effective way to study and comprehend the unique properties of hyperbolic geometry, which differ from those of Euclidean geometry. In addition to its utility in visualization, the Poincaré ball model allows for the application of standard Euclidean algorithms to perform geometric calculations in the hyperbolic plane, making it computationally practical. One of the primary applications of the Poincaré ball model is in computer graphics, particularly for representing the conformal structure of surfaces and analyzing complex datasets.

The Poincaré ball model allows for the depiction of the shape and curvature of a surface without regard to its size or position, making it a useful tool for analyzing and manipulating complex datasets in this field. The growing attention the Poincaré ball model has received in machine learning and data mining fields due to its ability to facilitate analysis and manipulation of large and complex datasets^33^. Eli et al.^40^ focus on the Poincaré ball model and use tangent space formalization to express classification problems, and the proposed algorithm provably converge and are highly scalable as they have complexities comparable to those of their Euclidean counterparts. They demonstrate superior performance accuracy on complex synthetic datasets and real-world datasets. Guo et al.^41^ proposed a Poincaré-based heterogeneous graph neural network for sequential recommendation, which models both sequential pattern information and hierarchical information.

The Poincaré ball model is a mathematical model that is used to represent hyperbolic geometry. It is a Riemannian manifold, denoted as$\left( {\mathbb {B}}_c^n, g^{{\mathbb {B}}_c}\right)$, where ${\mathbb {B}}_{c}^{n}=\left\{ u\in {{{\mathbb {R}}}^{n}}:\sqrt{c}\parallel u\parallel <1 \right\}$ is the open ball of radius $\frac{1}{\sqrt{c}}$ in -dimensional Euclidean space and $g^{{\mathbb {B}}_c}$ is the Riemannian metric defined as:

1 $$\begin{aligned} g_{u}^{{{{\mathbb {B}}}_{c}}}(\cdot ,\cdot )={{\left( \sigma _{u}^{c} \right) }^{2}}\langle \cdot ,\cdot \rangle =\frac{2}{1-c\parallel u{{\parallel }^{2}}}\langle \cdot ,\cdot \rangle , \end{aligned}$$

where $|\cdot |$ is the $l_2$ norm and $\langle \cdot , \cdot \rangle$ is the standard inner product. The curvature of the Poincaré ball model is determined by the value of *c*. When $c=0$, the Poincaré ball model reduces to Euclidean space, i.e. ${\mathbb {B}}_c^n = {\mathbb {R}}^n$. In this case, the Riemannian metric becomes the standard inner product, and the Poincaré ball model represents the familiar geometry of flat spaces. For $c>0$, the Poincaré ball model represents hyperbolic geometry, in which the curvature and radius is determined by the value of *c*. The Poincaré ball includes two fundamental operator operations: Möbius addition and scalar multiplication. These operations correspond to vector addition and scalar multiplication in Euclidean spaces, respectively. The Möbius addition is a non-commutative and non-associative operation that extends the concept of vector addition to the Poincaré ball, while scalar multiplication extends the concept of scalar multiplication from Euclidean spaces to the Poincaré ball. The Möbius addition ${\oplus }_{c}$ of $u,v\in {{{\mathbb {B}}}^{n}}$ is defined as:

2 $$\begin{aligned} u{{\oplus }_{c}}v=\frac{\left( 1+2c\langle u,v\rangle +c\parallel v{{\parallel }^{2}} \right) u+\left( 1-c\parallel u{{\parallel }^{2}} \right) v}{1+2c\langle u,v\rangle +{{c}^{2}}\parallel u{{\parallel }^{2}}\parallel v{{\parallel }^{2}}}. \end{aligned}$$

The operation $\oplus _c$ hold the following equalities: $u{{\oplus }_{c}}{\textbf{0}}={\textbf{0}}{{\oplus }_{c}}u=u$,$(-u{{\oplus }_{c}}u=u{{\oplus }_{c}}(-u)={\textbf{0}})$. Furthermore, the operation $\oplus _c$ recovers the Euclidean addition when c approaches zero, i.e. $c\rightarrow 0\Rightarrow u{{\oplus }_{c}}v\rightarrow u+v$. The Möbius scalar multiplication $\otimes _c$ of vector $u\in {\mathbb {B}}\backslash \{{\textbf{0}}\}$ by a scalar $v\in {\mathbb {R}}$ is defined according to:

3 $$\begin{aligned} v{{\otimes }_{c}}u=\frac{1}{\sqrt{c}}\text {tanh}(v\cdot \text {tan}{{\text {h}}^{\text {-1}}}(\sqrt{\text {c}}\parallel u\parallel ))\frac{u}{\parallel u\parallel }, \end{aligned}$$

and $v{{\otimes }_{c}}{\textbf{0}}={\textbf{0}}$. The Möbius scalar multiplication operation $\otimes _c$ converges to the standard Euclidean scalar multiplication as the scalar parameter *c* approaches zero. Mathematically, this can be represented as $c\rightarrow 0\Rightarrow v{{\otimes }_{c}}u=vu$. The distance function of $u,v\in {{{\mathbb {B}}}^{n}}$ in the Poincare model is given by:

4 $$\begin{aligned} {{d}_{c}}(u,v)=\frac{2}{\sqrt{c}}{{\tanh }^{-1}}\left( \sqrt{c}\left\| -u{{\oplus }_{c}}v \right\| \right) . \end{aligned}$$

Segmentation networks are typically designed to operate in Euclidean space. However, in order to execute segmentation within the Poincaré ball, it is required to establish a mapping from the Euclidean tangent space to the hyperbolic space. One way to achieve this is through the use of the exponential map, which projects a Euclidean vector onto the Poincaré ball with a fixed anchor point. For $p \in {\mathbb {D}}_c^n$, the exponential map $exp_p$: ${{T}_{p}}{\mathbb {D}}_{c}^{n}\rightarrow {\mathbb {D}}_{c}^{n}$ is given by:

5 $$\begin{aligned} {{\exp }_{p}}(v)=p{{\oplus }_{c}}\left( \tanh \left( \sqrt{c}\frac{\lambda _{\text {p}}^{c}\parallel v\parallel }{2} \right) \frac{v}{\sqrt{c}\parallel v\parallel } \right) . \end{aligned}$$

The exponential map is a mathematical function that can be used to map a tangent vector at a point in a manifold to a point on the manifold itself. This projection allows the segmentation network to operate effectively in the Poincaré ball while maintaining the geometric properties of the hyperbolic space. The above maps have more appealing forms, when $p=0$, namely for $v \in T_0 {\mathbb {D}}_c^n \backslash {{\textbf{0}}}, y \in {\mathbb {D}}_c^n \backslash {{\textbf{0}}}$:

6 $$\begin{aligned} {{\exp }_{0}}(v)=\tanh \left( \sqrt{c}\parallel v\parallel \right) \frac{v}{\sqrt{c}\parallel v\parallel }. \end{aligned}$$

### Hyperbolic embeddings

**Figure 1:**
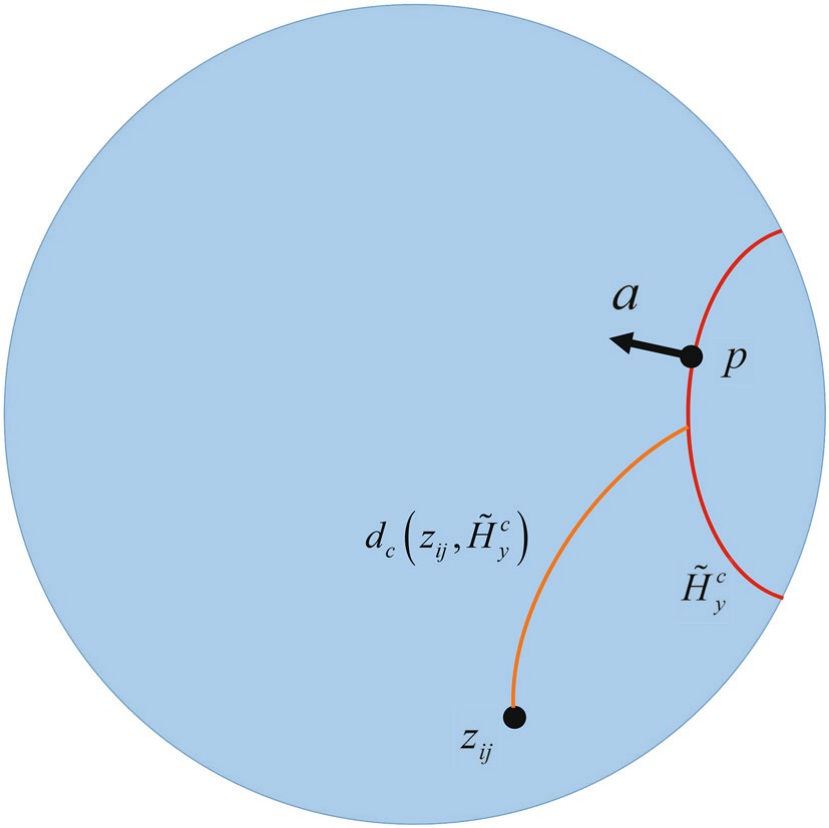
Visualization of the hyperbolic gyroplane ${\tilde{H}}_{y}^{c}$ and distance to output $z_{ij}$ on the manifold.

The problem of image segmentation involves the task of assigning a label to each pixel in an input image. The input RGB image is represented as $X \in {\mathbb {R}}^{w \times h \times 3}$, where *w* and *h* are the image’s width and height, respectively. A function $f(X): {\mathbb {R}}^{ w \times h \times 3} \rightarrow {\mathbb {R}}^{w \times h \times n}$ is used to transform each pixel in the input image to an *n*-dimensional representation matrix $Y \in {\mathbb {R}}^{w \times h \times n}$. A popular technique among contemporary methods for image classification is to process all pixels simultaneously by passing them through a linear layer and applying the softmax function to generate a *C*-dimensional probability distribution for each pixel across all *C* classes, i.e. $f(Y):{{{\mathbb {R}}}^{w\times h\times n}}\rightarrow {{{\mathbb {R}}}^{w\times h\times C}}$. The optimization of this approach is typically accomplished by using cross-entropy as the objective function. The pipeline design facilitates parallel processing of all pixels, thereby maximizing efficiency and optimizing the model through minimization of cross-entropy loss. The purpose of this study is to examine the application of hyperbolic space in the context of pixel-level classification for image segmentation. The gyroplane represents a hyperplane within the Poincaré ball, based on the geometric interpretation of hyperbolic multinomial logistic regression^42^. Specifically, for $p \in {\mathbb {D}}_c^n, a \in T_p {\mathbb {D}}_c^n \backslash {{\textbf{0}}}$, the Poincaré hyperplane is defined as:

7 $$\begin{aligned} {\tilde{H}}_{a,p}^{c}=\left\{ {{z}_{ij}}\in {\mathbb {D}}_{c}^{n}:\left\langle p{{\oplus }_{c}}{{z}_{ij}},a \right\rangle =0 \right\} , \end{aligned}$$

where $z_{ij} = exp_0(f(X)_{ij})$ denote the result of applying the exponential map to the neural network output at pixel location (*i*, *j*), $p \in {\mathbb {D}}_c^n$ is the reference point and $\text {a}\in {{T}_{p}}{\mathbb {D}}_{c}^{n}$ is the normal vector of the gyroplane. The set ${\tilde{H}}_{a, p}$ can also defined as the union of all images of geodesics in the hyperbolic space $D_c^n$ that are orthogonal to vector *a* and pass through the point *p* [27]. The hyperbolic distance between the point $z_{ij}$ and the gyroplane ${\tilde{H}}_{y}^{c}$ of class *y*, can be computed as Eq. (8). The Fig. 1 visualized the hyperbolic gyroplane ${\tilde{H}}_{y}^{c}$ and distance to output $z_{ij}$ on the manifold.

8 $$\begin{aligned} {{d}_{c}}\left( {{z}_{ij}},{\tilde{H}}_{y}^{c} \right) =\frac{1}{\sqrt{c}}{{\sinh }^{-1}}\left( \frac{2\sqrt{c}\left\langle {{p}_{y}}{{\oplus }_{c}}{{z}_{ij}},{{a}_{y}} \right\rangle }{\left( 1-c{{\left\| {{p}_{y}}{{\oplus }_{c}}{{z}_{ij}} \right\| }^{2}} \right) \left\| {{a}_{y}} \right\| } \right) . \end{aligned}$$

### Approximate treatment in hyperbolic space

The task of image segmentation necessitates simultaneous per-pixel classification. However, current implementations of hyperbolic multinomial logistic regression are computationally infeasible. To reduce the memory footprint of explicitly calculating the Möbius addition, we leverage an alternative computation for the margin likelihood that eliminates the need for explicit calculation of the Möbius addition. We overwrite the inner product in the numerator and squared norm in the denominator of Eq. (8). The overwrite of the inner product $\left\langle {{p}_{y}}{{\oplus }_{c}}{{z}_{ij}},{{a}_{y}} \right\rangle$ is defined as:

9 $$\begin{aligned} \langle {{p}_{y}}{{\oplus }_{c}}{{z}_{i}}j,{{a}_{y}}\rangle =\langle A{{p}_{y}}+B{{z}_{ij}},{{a}_{y}}\rangle =A\langle {{p}_{y}},a\rangle +B\langle {{z}_{ij}},a\rangle , \end{aligned}$$

where $$A=\frac{1+2c\langle {{p}_{y}},{{z}_{ij}}\rangle +c\Vert {{z}_{ij}}{{\Vert }^{2}}}{1+2c\langle {{p}_{y}},{{z}_{ij}}\rangle +{{c}^{2}}\Vert {{p}_{y}}{{\Vert }^{2}}\Vert {{z}_{ij}}{{\Vert }^{2}}}$$, $$B\text { }=\frac{1-c\Vert {{p}_{y}}{{\Vert }^{2}}}{1+2c\langle {{p}_{y}},{{z}_{ij}}\rangle +{{c}^{2}}\Vert {{p}_{y}}{{\Vert }^{2}}\Vert {{z}_{ij}}{{\Vert }^{2}}}$$.

The squared norm $\parallel p_y \oplus _c z_{ij}\parallel ^2$ of the Möbius addition can be performed efficiently utilizing the following method:

10 $$\begin{aligned} \Vert p_y \oplus _c z_{i j}\Vert ^2&=\sum _{m=1}^n(A p_y^m+B z_{i j}^m)^2 \end{aligned}$$

11 $$\begin{aligned}&=\sum _{m=1}^n(A p_y^m)^2+A p_y^m B z_{i j}^m+(B z_{i j}^m)^2 \end{aligned}$$

12 $$\begin{aligned}&=A^2 \sum _{m=1}^n(p_y^m)^2+2 A B \sum _{m=1}^n p_y^m z_{i j}^m+B^2 \sum _{m=1}^n(z_{i j}^m)^2 \end{aligned}$$

13 $$\begin{aligned}&=A^2\Vert p_y\Vert ^2+2 A B\langle p_y, z_{i j}\rangle +B^2\Vert z_{i j}\Vert ^2. \end{aligned}$$

Consequently, the logit of per pixel is computed as:

14 $$\begin{aligned} p\left( {\hat{y}}=y \mid z_{i j}\right) \propto \exp \left( \zeta _y\left( z_{i j}\right) \right) . \end{aligned}$$

The optimization of logit can be achieved through the implementation of the cross-entropy loss function and the gradient descent algorithm. Our method employs a approximation of the inner product and squared norm in the calculation of class logits, enabling the possibility of hyperbolic pixel-level classification. This novel approach effectively addresses the intractability previously encountered in current implementations of hyperbolic multinomial logistic regression, and enables more efficient per-pixel classification in parallel for image segmentation tasks.

## Model architecture

**Figure 2:**
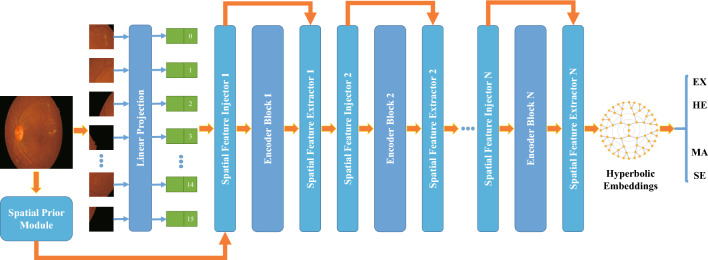
The overall architecture of the “VTA + HBE” model. The model uses a ViT network for feature extraction. A Spatial Prior Module enhances local continuity. Continuous features are input to the feature injector and extractor for interaction. Pixel-level classification employs hyperbolic embeddings, not Euclidean space.

The overall design of the proposed model is depicted in Fig. 2. The main components of the model, including the Vision Transformer for feature extraction and the integration of a spatial prior module to enhance image quality and capture local image continuity. Feature interaction is facilitated through the utilization of a spatial feature injector and extractor. Hyperbolic embeddings are employed for the final pixel-level classification of features.

### Vision transformer encoder

The ViT-Adapter design philosophy is a way of leveraging scalable NLP Transformer architectures for vision tasks^43^. The benefit of this straightforward design is that it allows us to leverage scalable NLP Transformer architectures and their efficient implementations almost immediately. The ViT-Adapter holds the potential to reconcile the discrepancy between ViT^44^ model and vision-specific model for segmentation task while maintaining the versatility of ViT, and could reap the benefits of advanced multi-modal pre-training techniques.

The Transformer receives 1D sequences of token embeddings as input. In order to adapt it for the processing of 2D medical images, we reshape the image $X \in {\mathbb {R}}^{w \times h \times 3}$ into a flattened 2D patches sequence $x_p \in {\mathbb {R}}^{N \times P^2 \times 3}$, where (*P*, *P*) is the resolution of each patch, $N=\frac{w\times h}{{{P}^{2}}}$ is the number of patches. The Transformer encoder subsequently comprises sequential layers of Multi-Headed Self-Attention (MSA) and Multi-Layer Perceptron (MLP) blocks. The MSA block is responsible for capturing the relationships between different elements in the input sequence. It does this by computing multiple attention heads in parallel, where each head learns a different aspect of the input sequence. The MLP block is responsible for applying a non-linear transformation to the output of the MSA block.

Layer Normalization (LN) is applied after each sub-layer in the Transformer encoder, including the MSA and MLP blocks. It normalizes the output of each sub-layer by subtracting its mean and dividing by its standard deviation, which improves the stability of the model during training and allows it to better generalize to new data. The residual function is added to the output of the sub-layer, which helps to mitigate the vanishing gradient problem and allows the model to learn deeper representations. Layer Normalization is implemented prior to each block, and residual connections are employed following each block. The ViT Encoder comprises a total of *L* layers, which can be mathematical represented as:

15 $$\begin{aligned} {\mathbf{y}}_{0} = & \left[ {{\mathbf{x}}_{{{\text{ class }}}} ;{\mathbf{x}}_{p}^{1} {\mathbf{E}};{\mathbf{x}}_{p}^{2} {\mathbf{E}}; \cdots ;{\mathbf{x}}_{p}^{N} {\mathbf{E}}} \right] + {\mathbf{E}}_{{pos}} ,\quad {\mathbf{E}} \in \mathbb{R}^{{P^{2} \cdot 3 \cdot D}} ,{\mathbf{E}}_{{pos}} \in \mathbb{R}^{{(N + 1) \cdot D}} \\ {\mathbf{y}}_{i}^{\prime } = & {\text{MSA}}\left( {{\text{LN}}\left( {{\mathbf{y}}_{{l - 1}} } \right)} \right) + {\mathbf{y}}_{{l - 1}} {\text{, }} \\ {\mathbf{y}}_{i} = & {\text{MLP}}\left( {{\text{LN}}\left( {{\mathbf{y}}_{l}^{\prime } } \right)} \right) + {\mathbf{y}}_{l}^{\prime } ,\quad i = 1 \cdots L \\ \end{aligned}$$

For image segmentation, we divide the encoder layers into uniform encoder block, and use the feature tokens from the each encoder block to feed into the vision transformer adapter module. The input image for the Transformer encoder is first processed through patch embeddings, where it is divided into non-overlapping $16 \times 16$ patches.

### Vision transformer adapter

#### Spatial prior module

**Figure 3:**
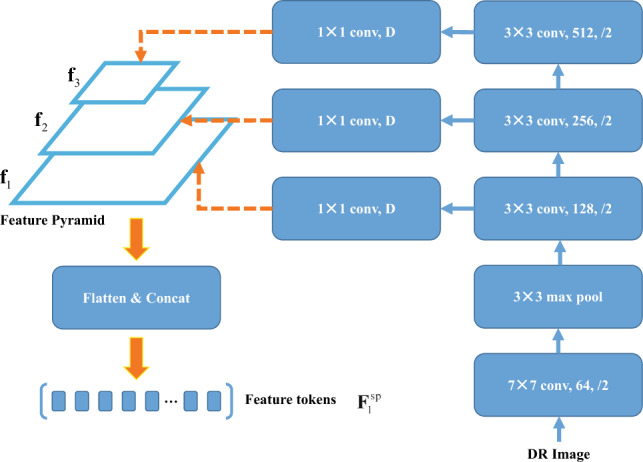
The architecture of the convolution-based Spatial Prior Modules.

Recent studies^45,46^ have demonstrated that utilizing convolutions with overlapping sliding windows can enhance the ability of transformers to effectively capture local continuity in input images. We present a novel addition to the Transformer encoder layer: the Spatial Prior Module (SPM), a convolution-based structure, which downsamples a $w \times h$ input image to various scales. The SPM module design architecture is shown in Fig. 3. The objective of this module is to concurrently model the local spatial contexts of images alongside the patch embeddings layer while preserving the integrity of the original architecture of the Vision Transformer.

We first feed the input image into the spatial prior module, and obtain a feature pyramid ${f_1, f_2, f_3}$, which contains *D*-dimensional feature maps (1024 dimension) with resolutions of 1/8, 1/16, and 1/32. Then we flatten and concatenate these feature maps into feature tokens ${\textbf{F}}_1^{\textrm{sp}} \in {\mathbb {R}}^{\left( \frac{H W}{8^2}+\frac{H W}{16^2}+\frac{H W}{32^2}\right) \times D}$ for feature interaction.

#### Spatial feature injector

The columnar structure of ViT results in single-scale and low-resolution feature maps, which negatively impact its performance in segmentation tasks relative to pyramid-structured transformers. To address this challenge, we propose the implementation of feature interaction modules, specifically the Spatial Feature Injector (SFI), to enhance communication between the adapter and ViT, thereby improving performance. We partition the transformer encoders of ViT into *N* equal blocks, the feature ${{\textbf{b}}_{i}}$ are from encoder block *i* of ViT model. To incorporate the spatial feature ${\textbf{F}}_{\text {i}}^{\text {sp}}$ into ${{\textbf{b}}_{i}}$, we employ multi-head cross-attention, which can be formulated as:

16 $$\begin{aligned} {{\textbf{b}}_{i}}={{\textbf{b}}_{i}}+{{\gamma }_{i}}\text {Attention}(\text {LN}({{{\textbf{F}}}_{\text {i}}}),\text {LN}({\textbf{F}}_{\text {i}}^{\text {sp}})), \end{aligned}$$

where ${\gamma }_{i}$ is a learnable parameter to modulate the balance the output of attention layer and the spatial feature ${\textbf{b}}_{i}$, which is initialized with a value of zero. The Pseudo-codes of the process of spatial feature injector are provided in Algorithm 1.
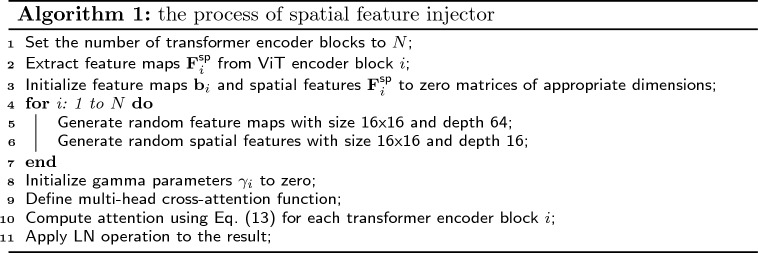


#### Spatial feature extractor

Upon incorporating the spatial feature via the SFI module, the output feature ${{{\textbf{b}}}_{i+1}}$ is obtained. Multi-scale feature extractor can enhance the spatial feature and extract the multi-scale feature. We employ a cross-attention layer to facilitate communication between the output feature ${{{\textbf{b}}}_{i+1}}$ and the spatial feature ${\textbf{F}}_{i}^{\text {sp}}$. Subsequently, a Convolutional Feed-Forward Network (CFFN) is introduced following the attention layer. Algorithm 2 shows the pseudo-codes of the process of spatial feature extractor, and the process are formulated as:

17 $$\begin{aligned} \widehat{{\textbf{F}}}_{i}^{\text {sp}}= & {} {\textbf{F}}_{i}^{\text {sp}}+\text {Attention}(\text {LN}({\textbf{F}}_{i}^{\text {sp}}),\text {LN}({{\textbf{b}}_{i+1}})) \nonumber \\ {\textbf{F}}_{i+1}^{\text {sp}}= & {} \widehat{{\textbf{F}}}_{i}^{\text {sp}}+\text {CFFN}(\text {LN}(\widehat{{\textbf{F}}}_{i}^{\text {sp}})). \end{aligned}$$

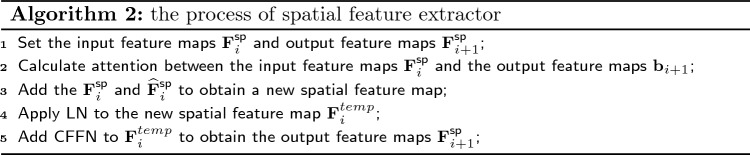


## Experiments

### Dataset

The IDRiD and DDR datasets are two important datasets in the field of retinal image segmentation, aimed at advancing the automatic diagnosis of diabetic retinopathy. Both datasets are important open resources for the development and evaluation of machine learning algorithms for diabetic retinopathy lesion detection and segementation.

IDRiD Dataset^47^ is a comprehensive resource for the segmentation and grading of retinal images, as part of the Retinal Image Challenge 2018. The dataset comprises 81 fundus images for the segmentation task, each with a resolution of 4288 × 2848 pixels. The images are accompanied by four pixel-level annotations for lesions of type EX (hard exudates), HE (haemorrhages), MA (microaneurysms), and SE (soft exudates), as applicable. Specifically, there are 81 EX annotations, 80 HE annotations, 81 MA annotations, and 40 SE annotations. The IDRiD dataset includes a training set with 54 images and a testing set with 27 images.

DDR Dataset^48^ offered by Ocular Disease Intelligent Recognition (ODIR-2019) provides support for lesion segmentation and detection and consists of 13,673 fundus images sourced from 147 healthcare institutions in 23 provinces in China. The segmentation task utilizes 757 fundus images, equipped with pixel-level annotations for EX, HE, MA, and SE lesions, totaling 486 EX, 601 HE, 570 MA, and 239 SE annotations. The dataset has been pre-partitioned into training (383 images), validation (149 images), and testing (225 images) subsets.

### Model configurations

Our model is designed to convert each pixel into an *n*-dimensional representation. In our experiments, the patch size of the ViT model is fixed at 16, *D*-dimension is set to 1024. The interaction times *N* is set to 4, which involves dividing the ViT encoder layers into 4 equal blocks for feature interaction. The width of ViT is set to 768, with a feed-forward network (FFN) size of 3072 and 12 heads. To reduce computational overhead, the ratio of the CFFN is set to 1/4, with a hidden size of 96 and the adapter has 12 heads.

### Implementation details

Our framework is implemented utilizing the PyTorch platform and executed on three NVIDIA GeForce RTX 3090Ti GPUs, each equipped with 24GB of memory. The newly integrated adapter modules have been randomly initialized and do not employ any pre-trained weights. The initial learning rate is set to 0.001. The model is trained for 160 epochs on the ADE20K dataset with a batch size of 2 and then fine-tuned on the IDRiD and DDR datasets. The optimization of Euclidean parameters is performed using Stochastic Gradient Descent (SGD) with a momentum of 0.9 and a polynomial learning rate decay of power 0.9. Hyperbolic parameters are optimized utilizing Riemannian Stochastic Gradient Descent (RSGD).

### Evaluation metrics

To evaluate the efficacy of the proposed model, the performance metrics used include the Area-Under-the-Curve (AUC) of the Precision-Recall (PR) curve and the Receiver Operating Characteristic (ROC) curve. These metrics have been widely adopted in previous research and competitions on fundus image segmentation. The assessment of the accuracy of true data in predictions is primarily conducted through the AUC_PR curve, while the performance of positively predicted data is evaluated using the AUC_ROC curve. Both the AUC_PR and AUC_ROC curves characterize the overall performance of different neural models.

### Experimental results

We trained DeepLab v3+^49^, UNet^50^, UNet++^51^ and Seg-B/16^52^ models on the IDRiD and DDR datasets using the model training parameters initialized according to the methods described in the corresponding original papers. Our VTA+HBE model was compared with these popular models. Table 1 displays the comparison results of the models on the IDRiD test set, showing that the VTA+HBE model achieved the highest performance in MA and SE segmentation predictions, and Seg-B/16’s performance in EX and HE segmentation predictions was comparable. Table 2 displays the comparison results of the models on the DDR test set, showing that the VTA+HBE model achieved the best performance in MA and EX segmentation predictions, but the EX and HE segmentation results were slightly inferior to those of the Seg-B/16 model.

**Table 1:** Performance comparison with widely-used segmentation methods on the IDRiD dataset.

Method	EX		HE		MA		SE	
	AUC_PR	AUC_ROC	AUC_PR	AUC_ROC	AUC_PR	AUC_ROC	AUC_PR	AUC_ROC
DeepLab v3+^49^	0.8293	0.9942	0.6389	0.9360	0.4024	0.9819	0.5968	0.9374
UNet^50^	0.8038	0.9903	0.6034	0.9431	0.4029	0.9820	0.5734	0.9102
UNet++^51^	0.8351	0.9940	0.6489	0.9499	0.4043	0.9820	0.5908	0.9326
Seg-B/16^52^	0.8601	0.9932	**0**.**6504**	**0**.**9503**	**0**.**4068**	**0**.**9849**	0.5849	0.9378
VTA+HBE (Ours)	**0**.**8640**	**0**.**9943**	0.6494	0.9487	0.4063	0.9839	**0**.**5978**	**0**.**9463**

Bold font is used to indicate the best result compared to other models.

**Table 2:** Performance comparison with widely-used segmentation methods on the DDR dataset.

Method	EX		HE		MA		SE	
	AUC_PR	AUC_ROC	AUC_PR	AUC_ROC	AUC_PR	AUC_ROC	AUC_PR	AUC_ROC
DeepLab v3+^49^	0.5532	0.9730	0.3782	0.9312	0.0334	0.9212	0.2190	0.8671
UNet^50^	0.5512	0.9619	0.3727	0.9392	0.0365	0.9378	0.2434	0.8756
UNet++^51^	0.5579	0.9723	0.3880	0.9398	0.0443	0.9245	0.2453	0.8767
Seg-B/16^52^	0.5435	**0**.**9747**	**0**.**3930**	0.9367	0.0923	0.9276	0.2687	0.8578
VTA+HBE (Ours)	**0**.**5607**	0.9745	0.3778	**0**.**9403**	**0**.**1066**	**0**.**9409**	**0**.**2694**	**0**.**8857**

Bold font is used to indicate the best result compared to other models.

### Ablation studies

We employ the vision transformer with transposed convolution as our baseline, in which the feature sequence generated by each coding block is reshaped, then subjected to upsampling via transposed convolution. The resulting upsampled feature matrix is then reshaped and utilized as the input feature sequence for the next coding block. This baseline preserves the dimensions and size of each feature sequence in our model. We enhanced this baseline with our proposed VTA technique and named the resulting model as “baseline+VTA”. Further, we added (HBE) to classify the feature matrix at the pixel level, resulting in the “VTA + HBE” model.

**Figure 4:**
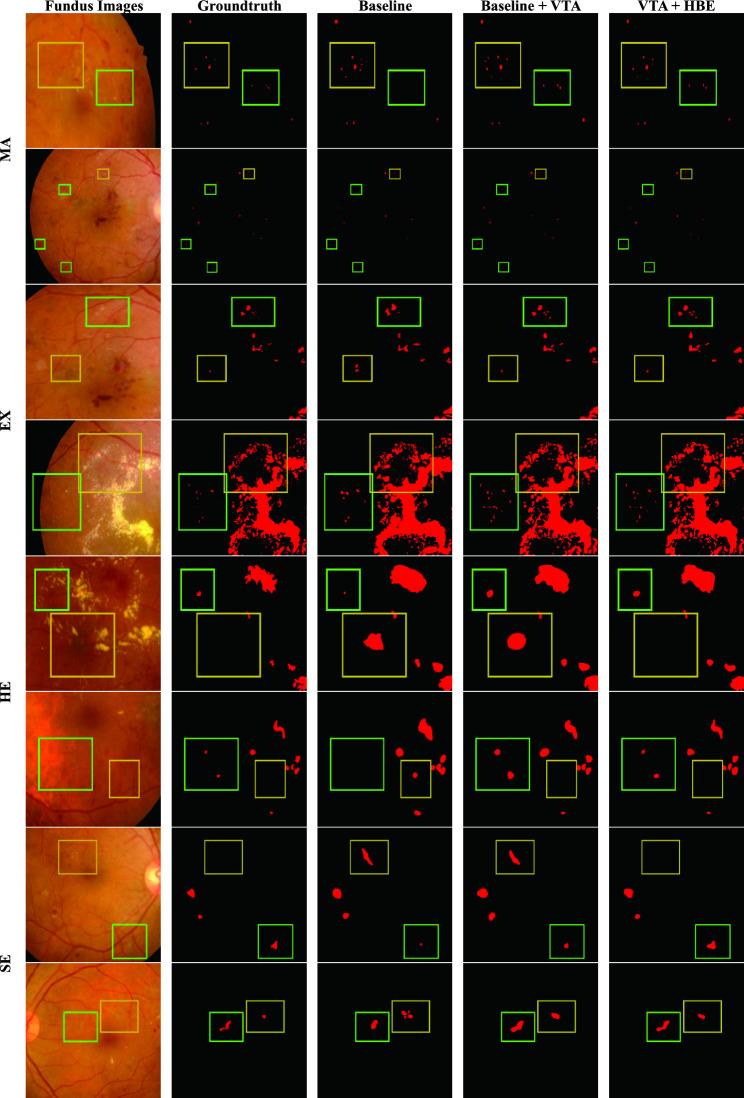
Visualization of the efficacy of the VTA and the HBE. We randomly selected and visualized predictions from the IDRiD and DDR test sets. The green and yellow boxes highlight the areas where DR segmentation was improved by the VTA and the HBE, respectively. As can be seen from the figure, the HBE enhances finer segmentation predictions, while the VTA prevents certain misclassification predictions.

To demonstrate the efficacy of VTA and HBE, we randomly selected and visualized predictions from the IDRiD and DDR test sets. Figure 4 presents a comparison of the segmentation results with the original images and ground truths. Segmentation plots of the baseline, baseline + VTA, and VTA + HBE models were used to show the improvement of each component of our network. The green and yellow boxes highlight the areas where DR segmentation was improved by the VTA and the HBE, respectively. As evident from the figure, HBE enhances finer segmentation predictions, while VTA prevents certain misclassification predictions. Tables 3 and 4 compare the performance of the baseline, baseline + VTA, and VTA + HBE models on the IDRiD and DDR test sets. The results demonstrate that both VTA and HBE have a positive influence on model accuracy, with VTA showing significant improvement in all segmentation accuracy metrics. To further analyze the effectiveness of HE, the curvature value were fine-tuned and trained on the IDRiD and DDR dataset, and the model performance is presented in Tables 5 and 6. As shown in the table, the performance of the model improves to some extent with an increase in the curvature value, but performance decreases rapidly when the curvature value is greater than 2.

**Table 3:** Performance comparison of ablation studies on the IDRiD dataset.

Ablation studies	EX		HE		MA		SE	
	AUC_PR	AUC_ROC	AUC_PR	AUC_ROC	AUC_PR	AUC_ROC	AUC_PR	AUC_ROC
Baseline	0.7934	0.8956	0.5931	0.9137	0.3823	0.9423	0.5130	0.9024
Baseline + VTA	0.8603	0.9889	0.6486	0.9497	0.4050	0.9732	0.5903	0.9316
VTA+HBE	**0**.**8640**	**0**.**9943**	**0**.**6494**	**0**.**9487**	**0**.**4063**	**0**.**9839**	**0**.**5978**	**0**.**9463**

Bold font is used to indicate the best result compared to other models.

**Table 4:** Performance comparison of ablation studies on the DDR dataset.

Ablation studies	EX		HE		MA		SE	
	AUC_PR	AUC_ROC	AUC_PR	AUC_ROC	AUC_PR	AUC_ROC	AUC_PR	AUC_ROC
Baseline	0.4923	0.9081	0.3248	0.8748	0.0830	0.9021	0.2215	0.8353
Baseline + VTA	0.5598	0.9635	0.3739	0.9392	0.1023	0.9382	0.2594	0.8748
VTA+HBE	**0**.**5607**	**0**.**9745**	**0**.**3778**	**0**.**9403**	**0**.**1066**	**0**.**9409**	**0**.**2694**	**0**.**8857**

Bold font is used to indicate the best result compared to other models.

**Table 5:** Performance comparison of different curvature value on the IDRiD dataset.

Curvature value	EX		HE		MA		SE	
	AUC_PR	AUC_ROC	AUC_PR	AUC_ROC	AUC_PR	AUC_ROC	AUC_PR	AUC_ROC
0	0.8623	0.9887	0.6465	0.9354	0.4009	0.9712	0.5909	0.9303
0.2	0.8643	0.9892	0.6459	0.9332	0.4023	0.9729	0.5912	0.9392
0.4	0.8620	0.9905	0.6485	0.9367	0.4034	0.9829	0.5923	0.9402
0.8	**0**.**8659**	**0**.**9948**	0.6487	0.9459	0.4023	0.9810	0.5923	0.9434
1.0	0.8640	0.9943	**0**.**6494**	0.9487	**0**.**4063**	**0**.**9839**	**0**.**5978**	**0**.**9463**
2.0	0.8603	0.9932	0.6492	**0**.**9503**	0.4021	0.9804	0.5923	0.9320
5.0	0.8610	0.9837	0.6421	0.9358	0.4002	0.9723	0.5938	0.9335
10.0	0.7423	0.8729	0.5293	0.8195	0.3474	0.8538	0.5231	0.9025

Bold font is used to indicate the best result compared to other models.

**Table 6:** Performance comparison of different curvature value on the DDR dataset.

Curvature value	EX		HE		MA		SE	
	AUC_PR	AUC_ROC	AUC_PR	AUC_ROC	AUC_PR	AUC_ROC	AUC_PR	AUC_ROC
0	0.5542	0.9710	0.3720	0.9227	0.0946	0.9203	0.2621	0.8759
0.2	0.5578	0.9732	0.3750	0.9312	0.1043	0.9401	0.2602	0.8809
0.4	0.5592	0.9741	0.3729	0.9376	0.1051	0.9372	0.2598	0.8840
0.8	0.5594	0.9720	0.3769	**0**.**9473**	0.1037	0.9391	0.2635	0.8824
1.0	**0**.**5607**	0.9745	0.3778	0.9403	**0**.**1066**	**0**.**9409**	**0**.**2694**	**0**.**8857**
2.0	0.5602	**0**.**9775**	**0**.**3784**	0.9326	0.1021	0.9391	0.2603	0.8821
5.0	0.5532	0.9707	0.3742	0.9340	0.1003	0.9289	0.2583	0.8723
10.0	0.5228	0.9532	0.3494	0.9153	0.0845	0.9102	0.2226	0.7693

Bold font is used to indicate the best result compared to other models.

## Conclusions

The main contribution of this study is the introduction of a hyperbolic space-based Transformer model architecture for DR image segmentation. The VTA+HBE model adapts the original Vision Transformer model by adding a spatial prior module that leverages convolutional neural networks to extract image features. This allows the model to capture both spatial and semantic information, which is crucial for the accurate segmentation of lesions in DR. The VTA+HBE model also incorporates a spatial feature injector and extractor to improve feature interaction. The hyperbolic embeddings for pixel-level classification addresses the challenges faced by current implementations of hyperbolic polynomial logistic regression, resulting in more efficient and accurate segmentation of lesions in DR.

Through extensive experimentation, we provide compelling evidence of the efficacy of the proposed Transformer-based model architecture in extracting meaningful features from DR images, while also demonstrating the potential benefits of incorporating hyperbolic space within deep learning frameworks. Given the severe consequences of DR, including vision impairment and blindness, the research community has shown a growing interest in developing automated DR detection systems. Therefore, the significance of this study lies in its potential to aid in the accurate diagnosis of DR by automating the detection of DR lesions.

In future research, we will delve deeper into exploring the synergistic integration of hyperbolic space and diverse deep learning architectures, with a specific focus on their application in DR classification and segmentation tasks. We aim to advance the field by investigating novel techniques and methodologies that leverage the unique properties of hyperbolic space to enhance the performance, interpretability, and generalization capabilities of deep learning models when applied to DR classification or segmentation tasks.

## Data Availability

The datasets used and/or analysed during the current study available from the corresponding author on reasonable request.
